# Discovery of a Thermostable Nigerose Phosphorylase for the Efficient Chemoenzymatic Radiosynthesis of a *S. aureus*–Targeted ^18^F-Disaccharide

**DOI:** 10.2967/jnumed.125.271829

**Published:** 2026-08

**Authors:** Jung Min Kim, Sang Hee Lee, Shari Dhaene, Adolfo Ancona, Jaelim Kim, Marina López-Álvarez, Alexandre M. Sorlin, Joseph Blecha, Wenxing Zou, Kiel D. Neumann, Giuseppe Felice Mangiatordi, Eunsung Lee, Bernd Nidetky, Robert R. Flavell, Youngho Seo, Joanne Engel, Michael A. Ohliger, Tom Desmet, David M. Wilson

**Affiliations:** 1 Department of Radiology and Biomedical Imaging, University of California, San Francisco, San Francisco, California;; 2 Department of Biotechnology, Ghent University, Gent, Belgium;; 3 CNR-Istituto di Cristallografia, Bari, Italy;; 4 Department of Biotechnology, Chemistry and Pharmacy, University of Siena, Siena, Italy;; 5 Department of Chemistry, Pohang University of Science and Technology, Pohang, Republic of Korea;; 6 Department of Radiology, St. Jude Children’s Research Hospital, Memphis, Tennessee;; 7 Department of Chemistry, Seoul National University, Seoul, Republic of Korea;; 8 Institute of Biotechnology and Biochemical Engineering, Graz University of Technology, Graz, Austria;; 9 Department of Nuclear Engineering, University of California, Berkeley, California;; 10 UCSF Helen Diller Family Comprehensive Cancer Center, San Francisco, California;; 11 Department of Medicine, University of California, San Francisco, San Francisco, California; and; 12 Department of Radiology, Zuckerberg San Francisco General Hospital, San Francisco, California

**Keywords:** infection imaging, PET, *Staphylococcus aureus*, ^18^F-sakebiose, nigerose phosphorylase

## Abstract

*Staphylococcus aureus* is a leading cause of life-threatening infections worldwide. The diagnosis and treatment of *S. aureus* infections are further complicated by the global rise of antimicrobial resistance. Therefore, rapid detection of active *S. aureus* remains a critical unmet need to provide effective infection management. In this study, we identified 2-deoxy-2-[^18^F]-fluorosakebiose ([^18^F]FSK) as an optimal radiotracer to detect active *S. aureus* and established its efficient chemoenzymatic radiosynthesis to facilitate clinical translation. **Methods**: Several [^18^F]FDG-derived disaccharides were obtained via reverse phosphorolysis: [^18^F]FSK (α-1,3–linked), 2-deoxy-[^18^F]-fluoromaltose (α-1,4–linked), 2-deoxy-2-[^18^F]-fluorolaminaribiose (β-1,3–linked), and 2-deoxy-2-[^18^F]-fluorocellobiose (β-1,4–linked). These tracers were screened in vitro in multiple *S. aureus* isolates to identify bacterial incorporation. The lead candidate, [^18^F]FSK, was further characterized via biodistribution and dosimetry analyses and evaluated in a *S. aureus* myositis model to assess antimicrobial treatment response. Finally, to promote the clinical translation of [^18^F]FSK, 2 different radiosynthetic strategies were investigated: reverse phosphorolysis of [^18^F]FDG using maltose phosphorylase and using newly identified nigerose (also called sakebiose) phosphorylases. **Results**: Nigerose phosphorylase–derived [^18^F]FSK was selected as the optimal radiotracer for detecting *S. aureus* because of its consistent and robust uptake in multiple *S. aureus* isolates. [^18^F]FSK demonstrated favorable distribution and elimination over time, with minimal nonspecific signals in uninfected organs. The estimated human effective doses indicated an effective dose comparable to that of [^18^F]FDG. The radiosynthesis of [^18^F]FSK, initially obtained as an accidental byproduct of maltose phosphorylase catalysis, was further improved using a nigerose phosphorylase originating from thermostable *Spirochaeta thermophila*, enabling nearly quantitative conversion of [^18^F]FDG to [^18^F]FSK (up to 97%). **Conclusion**: We demonstrated that [^18^F]FSK is a potent and robust PET radiotracer for the detection of active *S. aureus* in vivo and aids in the selection of an appropriate antimicrobial treatment. These findings highlight the potential use of [^18^F]FSK in promoting the effective management of *S. aureus* infections in clinical settings.

## Visual Abstract

**Figure:**
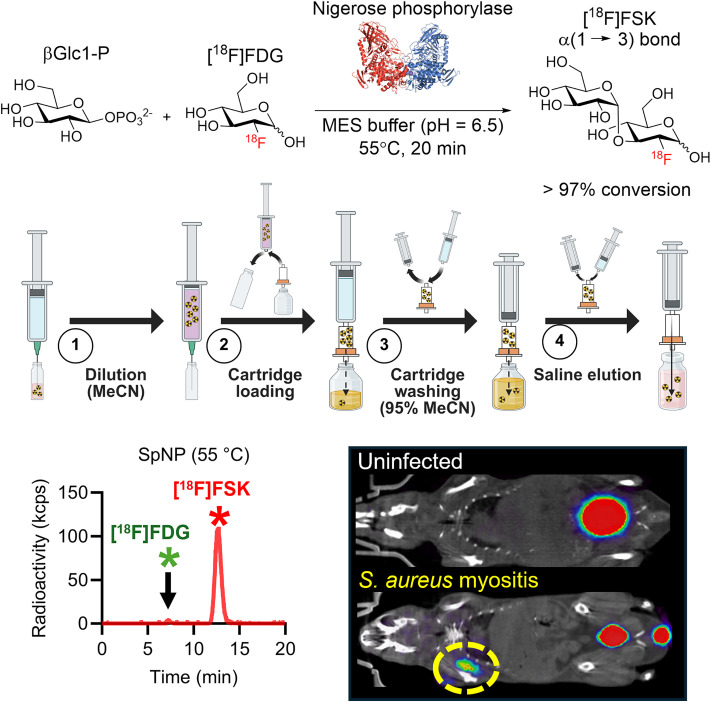

Bacterial infections were the second leading cause of death worldwide in 2019, and their diagnosis and treatment are increasingly complicated by the global rise of antimicrobial resistance (1). *Staphylococcus aureus* is a leading cause of invasive and potentially deadly infections, including bacteremia (2), endocarditis (3), osteomyelitis (4), and implant-associated infections (5). Notably, methicillin-resistant *S. aureus* (MRSA) has been listed by the World Health Organization as a high-priority bacterial pathogen (6).

Early identification of *S. aureus* infections is essential for guiding effective treatment, improving clinical outcomes, and reducing the risk of complications and recurrence. Although CT and MRI offer high-resolution structural information associated with infectious and inflammatory processes (7), their diagnostic utility relies on the detection of morphologic abnormalities (e.g., bone destruction), the formation of abscesses, and edema, which are secondary structural changes (8). [^18^F]FDG PET reports metabolic information by detecting abnormal glycolytic activity of activated immune cells, thereby improving sensitivity for identifying inflammatory responses across a wide range of diseases, including infections and malignancies. However, [^18^F]FDG PET cannot readily differentiate infection from inflammation and is often not optimal for early diagnosis and therapeutic response monitoring, potentially contributing to antibiotic overuse (9).

Despite its limitations as a radiotracer for identifying living microorganisms, [^18^F]FDG has historically served as a practical precursor to generate ^18^F-labeled glycosylated products, particularly because it is produced in cyclotron centers worldwide and is therefore widely available (10). This approach has been extended to the development of tracers targeting bacteria-specific sugar or sugar alcohol metabolism in diverse microorganisms. In 2014, the sorbitol analog ^18^F-fluorodeoxysorbitol ([^18^F]FDS), which is readily available via simple reduction from [^18^F]FDG, was reported as a radiotracer targeting sorbitol metabolism in Enterobacteriaceae but not in mammalian cells and gram-positive pathogens, including *S. aureus* (11).

To expand the scope of [^18^F]FDG-derived tracers for “on-demand” radiosynthesis of pathogen-specific PET radiotracers, we previously reported 1-step chemoenzymatic radiosyntheses of ^18^F-labeled disaccharides using reverse phosphorolysis (12). ^18^F-labeled disaccharides were synthesized using their corresponding phosphorylase enzymes, such as ^18^F-fluordeoxymaltose ([^18^F]FDM; α-1,4–linked) via maltose phosphorylase (MP). Interestingly, MP produces [^18^F]FDM as the major product, while also generating ^18^F-fluorosakebiose ([^18^F]FSK; α-1,3–linked) as a minor product. In our preliminary analysis of α-linked tracers, [^18^F]FDM and [^18^F]FSK showed comparable uptake in *S. aureus* (ATCC 12600), whereas the trehalose derivative ^18^F-fluorodeoxytrehalose (α-1,1–linked) showed markedly lower sensitivity to this organism, consistent with previous reports describing its specificity for mycobacteria (13). Although the β-linked tracers were not tested in our previous study, the cellobiose-derivative ^18^F-fluorodeoxycellobiose ([^18^F]FDC; β-1,4–linked) has been reported to show uptake in *S. aureus* (14). The bacterial specificity of the laminaribiose derivative ^18^F-fluorodeoxylaminaribiose ([^18^F]FDL; β-1,4–linked) remains unknown.

In the current study, we sought to identify an optimal [^18^F]FDG-derived disaccharide PET tracer for the robust detection of *S. aureus* and found that [^18^F]FSK is a particularly promising candidate. The radiochemical yield of [^18^F]FSK was further improved using a nigerose phosphorylase (NP) originating from thermostable *Spirochaeta thermophila* (SpNP). Collectively, these efforts establish [^18^F]FSK as a robust and translationally feasible *S. aureus*–sensitive probe, setting the stage for the first human application of a chemoenzymatically derived PET tracer.

## MATERIALS AND METHODS

Condensed methods are reported here. Detailed experimental procedures are described in the supplemental materials, available at http://jnm.snmjournals.org (15–32). All protocols were approved by the University of California, San Francisco Biosafety, Radiation Safety, and Animal Care and Use Committees. ^18^F-labeled disaccharides were prepared in accordance with our established protocol (12) for in vitro or in vivo studies. [^18^F]FDS was synthesized using NaBH_4_ (33). For in vivo studies, [^18^F]FSK (∼5.5 MBq) was formulated in saline containing voglibose (1 mg per mouse) and injected via the tail vein of female CBA/J mice (age 9–11 wk).

### In Vitro Assay

^18^F-labeled tracers were screened in vitro in accordance with our established protocol (16). Briefly, the bacterial cultures were incubated with ^18^F-labeled tracers (final concentration, 0.1 MBq/mL) at 37 °C for 90 min. Uptake of radioactivity in pellets and supernatants was measured using a γ-counter.

### PET/CT Data Acquisition

Small-animal PET/CT scans were performed on a nanoScan PET123S/CT1512 system (Mediso). The data were corrected for decay, dead time, and random coincidences and reconstructed into a 160 × 160 × 184 matrix with a voxel size of 0.8 mm via a 3-dimensional ordered-subsets expectation maximization algorithm using manufacturer-provided software. Attenuation and scatter corrections were applied using the coregistered CT.

### Murine Myositis Model

Ten mice were inoculated with MRSA (1 × 10^6^ colony-forming units) intramuscularly into the left deltoid, as previously described (16).

### Enzyme Preparation

All genes were obtained via GeneArt (Thermo Fisher Scientific) and cloned in an inducible expression vector (pET21a).

### Radiosynthesis of [^18^F]FDG-Derived Disaccharides

Chemoenzymatic radiosyntheses of ^18^F-labeled disaccharides (both α- and β-linked) were conducted as previously reported (12).

### Statistical Analysis

All statistical analyses and graph generation were performed using Prism version 10 (GraphPad Software). *P* values of less than 0.05 were considered statistically significant. All data were expressed as the mean ± SD, unless otherwise specified.

## RESULTS

### Identification of [^18^F]FSK as an Optimal [^18^F]FDG-Derived Disaccharide for Detecting *S. aureus*

**FIGURE 1.**
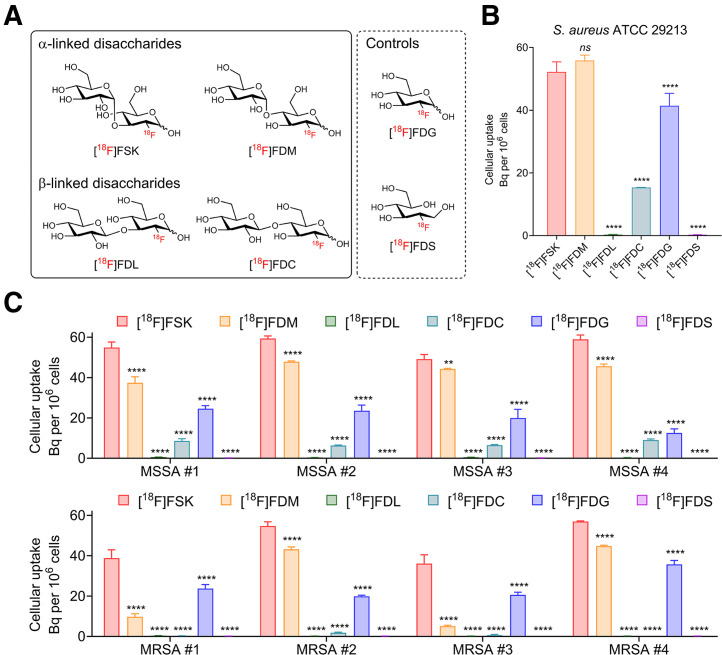
[^18^F]FSK shows high uptake in *S. aureus*. (A) Chemical structures of ^18^F-disaccharides and [^18^F]FDG/[^18^F]FDS (controls). Comparison of in vitro uptake of ^18^F-tracers in *S. aureus* ATCC 29213 (B) and clinical isolates of methicillin-susceptible *S. aureus* (MSSA) and MRSA (C). Data were analyzed using 1-way ANOVA with Dunnett multiple comparison test. **P < 0.01; ***P < 0.001; ****P < 0.0001; ns = not significant.

To identify an optimal ^18^F-labeled disaccharide for *S. aureus* detection, we initially tested 2 α-linked analogs ([^18^F]FSK and [^18^F]FDM) and 2 β-linked analogs ([^18^F]FDC and [^18^F]FDL) in vitro in *S. aureus* (ATCC 29213) (Figs. 1A and 1B). Uptake of [^18^F]FSK in a laboratory strain of *S. aureus* was comparable to that of [^18^F]FDM. In contrast, [^18^F]FDC showed low uptake in *S. aureus* compared with the α-linked analogs, whereas no detectable uptake was observed for [^18^F]FDL, similar to the negative control [^18^F]FDS. We further tested the uptake of ^18^F-labeled disaccharides in methicillin-susceptible *S. aureus* and MRSA clinical isolates (Fig. 1C). Uptake of [^18^F]FSK was robust in all bacteria studied, whereas uptake of [^18^F]FDM was significantly lower than that of [^18^F]FSK and inconsistent across isolates. Collectively, these results demonstrated that [^18^F]FSK was a highly sensitive ^18^F-labeled disaccharide for detecting *S. aureus*. Additional evaluation of [^18^F]FSK and [^18^F]FDM in other bacterial species, including laboratory strains and clinical isolates of *Staphylococcus epidermidis, Streptococcus agalactiae, Streptococcus pyogenes*, and *Escherichia coli*, revealed higher uptake of [^18^F]FSK in *S. epidermidis* but lower uptake in *Streptococcus* species and *E. coli* versus [^18^F]FDM (Supplemental Fig. 1).

### Biodistribution and Dosimetry Analysis of [^18^F]FSK in Uninfected Mice

**FIGURE 2.**
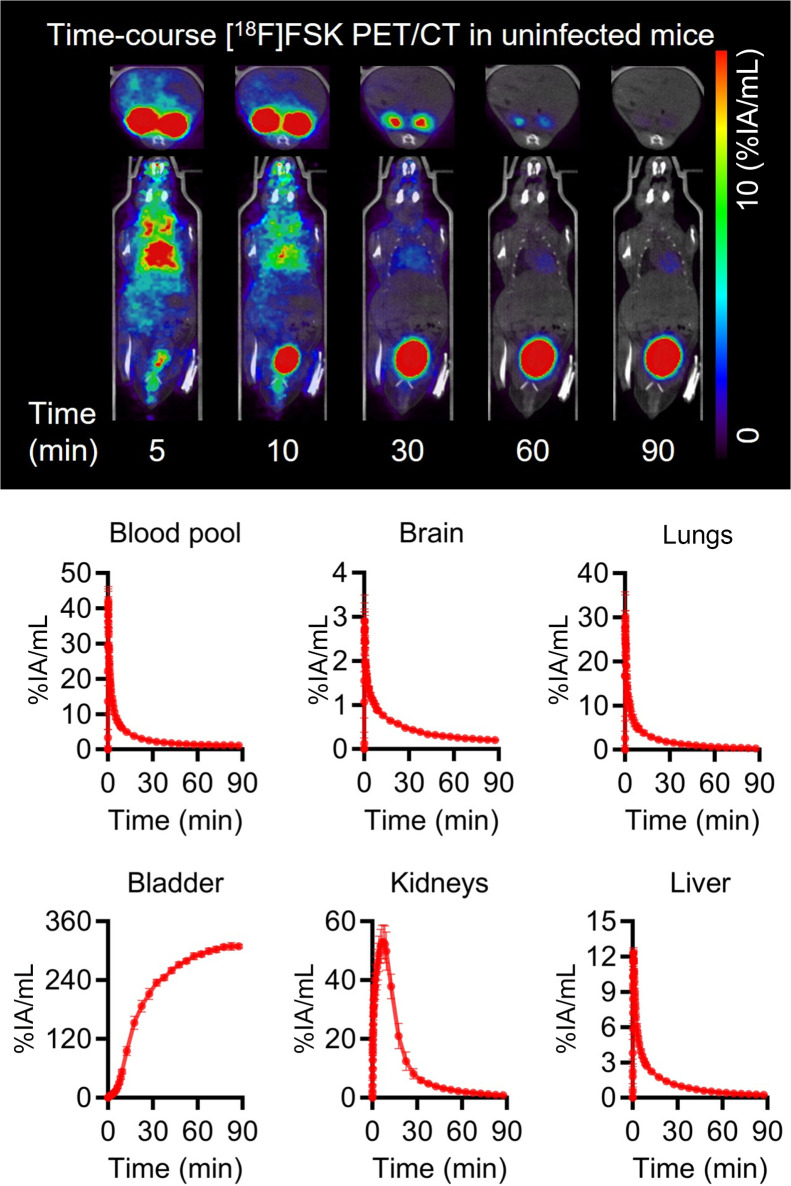
Dynamic small-animal PET/CT biodistribution analysis of [^18^F]FSK in uninfected mice. Time–activity curves are expressed as mean ± SEM.

To characterize the distribution and elimination profile of [^18^F]FSK in vivo, we performed dynamic small-animal PET/CT imaging of [^18^F]FSK in 4 uninfected mice (Fig. 2). Time–activity curves of [^18^F]FSK in major organs revealed rapid blood clearance and predominant renal excretion. After peak uptake in the liver, [^18^F]FSK rapidly washed out, suggesting minimal nonspecific accumulation in the liver and gastrointestinal tract, also supported by ex vivo analysis (Supplemental Fig. 2). The area under the curve values for the liver, kidneys, and bladder suggest that urinary excretion was the predominant clearance route (Table 1). Next, we performed human dosimetry for [^18^F]FSK by extrapolating mouse distribution data (4 males, 4 females) using International Commission on Radiological Protection (ICRP) 60 and 103 models, summarized in Supplemental Tables 1–4. The urinary bladder wall was estimated to receive the highest absorbed dose in both male and female mice. The estimated effective doses were 0.013 ± 0.001 and 0.020 ± 0.008 mSv/MBq in male and female mice, respectively, using ICRP 103 tissue-weighting factors, with corresponding estimates of 0.017 and 0.029 mSv/MBq using ICRP 60 tissue-weighting factors.

**TABLE 1.** Pharmacokinetic Parameters of [^18^F]FSK Derived from Time–Activity Curves

Parameter	Blood pool	Brain	Lung	Liver	Kidneys	Bladder
C_max_ (%IA/mL)	49.0 ± 4.4	3.8 ± 0.6	36.9 ± 6.1	12.6 ± 0.7	53.3 ± 11.8	313.1 ± 11.2
*t*_max_ (min)	0.3 ± 0.1	0.4 ± 0.1	0.3 ± 0.1	0.6 ± 0.1	7.3 ± 1.0	87.5 ± 0.0
AUC (%IA/mL·min)	281.1 ± 23.9	41.5 ± 3.7	190.1 ± 43.7	112.6 ± 8.2	1011.0 ± 171.5	19615.3 ± 1127.1
*t*_1/2α_ (min)	1.42	2.50	1.23	1.64	ND	ND
*t*_1/2β_ (min)	23.61	38.17	21.5	22.70	18.34	ND

ND = not determined.

### [^18^F]FSK PET Can Identify Therapeutic Success Versus Failure in a Preclinical Model of MRSA Infection

**FIGURE 3.**
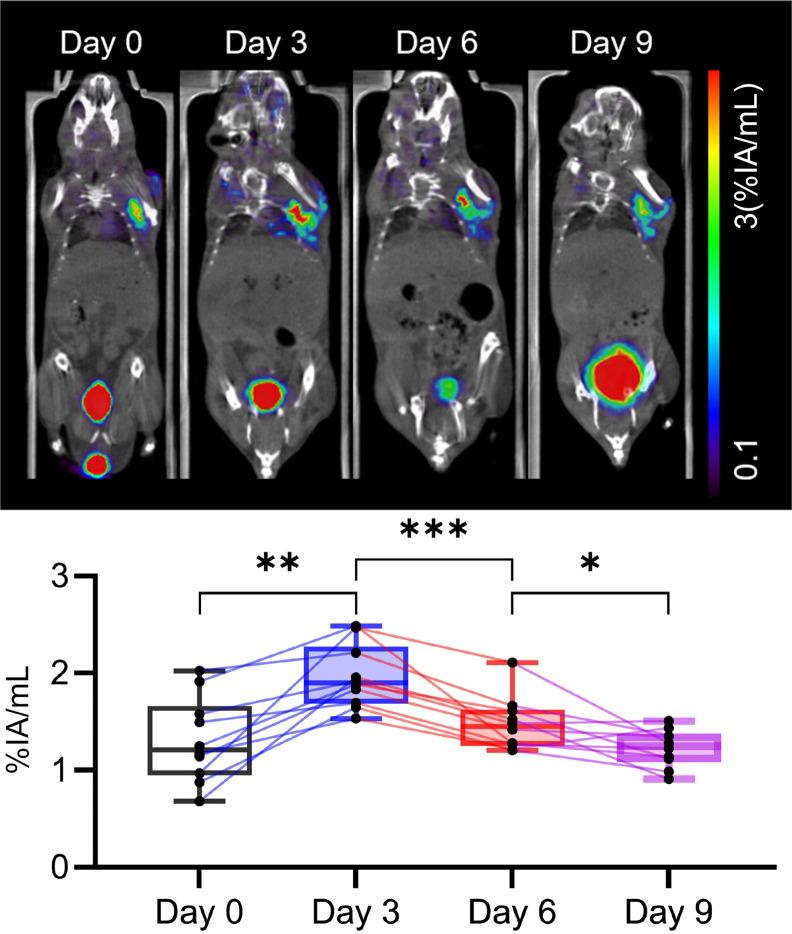
Representative longitudinal [^18^F]FSK small-animal PET/CT images of MRSA-inoculated mice acquired at days 0, 3, 6, and 9. Region of interest analysis of [^18^F]FSK at infected tissue over time. Data were analyzed using paired Student *t* test; *P < 0.05, **P < 0.01, ***P < 0.001.

To assess the feasibility of [^18^F]FSK PET for guiding appropriate antibiotic therapy, 10 mice inoculated with MRSA (minimum inhibitory concentration of >16 μg/mL for oxacillin and ≤0.5 μg/mL for vancomycin) in the left deltoid were initially treated with oxacillin (days 0–2; 100 mg/kg intravenously every 12 h; total of 6 doses), followed by vancomycin (days 3–9; 100 mg/kg intravenously every 24 h, total of 6 doses) (Supplemental Fig. 3). To follow antimicrobial response during treatment, [^18^F]FSK PET scans were conducted on days 0 (before treatment), 3, 6, and 9 (Fig. 3). As expected, treatment of MRSA with oxacillin was not effective. The [^18^F]FSK signal in the MRSA-inoculated sites significantly increased between days 0 and 3 (*P* < 0.01), indicating progressive MRSA infection when treating it with a β-lactam antibiotic. The antibiotic was switched to vancomycin on day 3, leading to a significant reduction of [^18^F]FSK signal at the MRSA-inoculated site on days 6 (*P* < 0.001) and 9 (*P* < 0.05). In contrast, the control group (*n* = 6), which received only oxacillin, also showed an initial progressive increase in [^18^F]FSK signal between days 0 and 3 (Supplemental Fig. 4). No imaging of this group was performed beyond day 3 because the experimental endpoint criteria were met (requiring euthanasia) between days 5 and 6. Overall, the results highlighted the potential of [^18^F]FSK to monitor therapeutic efficacy and thereby guide appropriate therapy in MRSA infection.

### Improved Yield of [^18^F]FSK Obtained Using MP in Presence of Inorganic Phosphate

**FIGURE 4.**
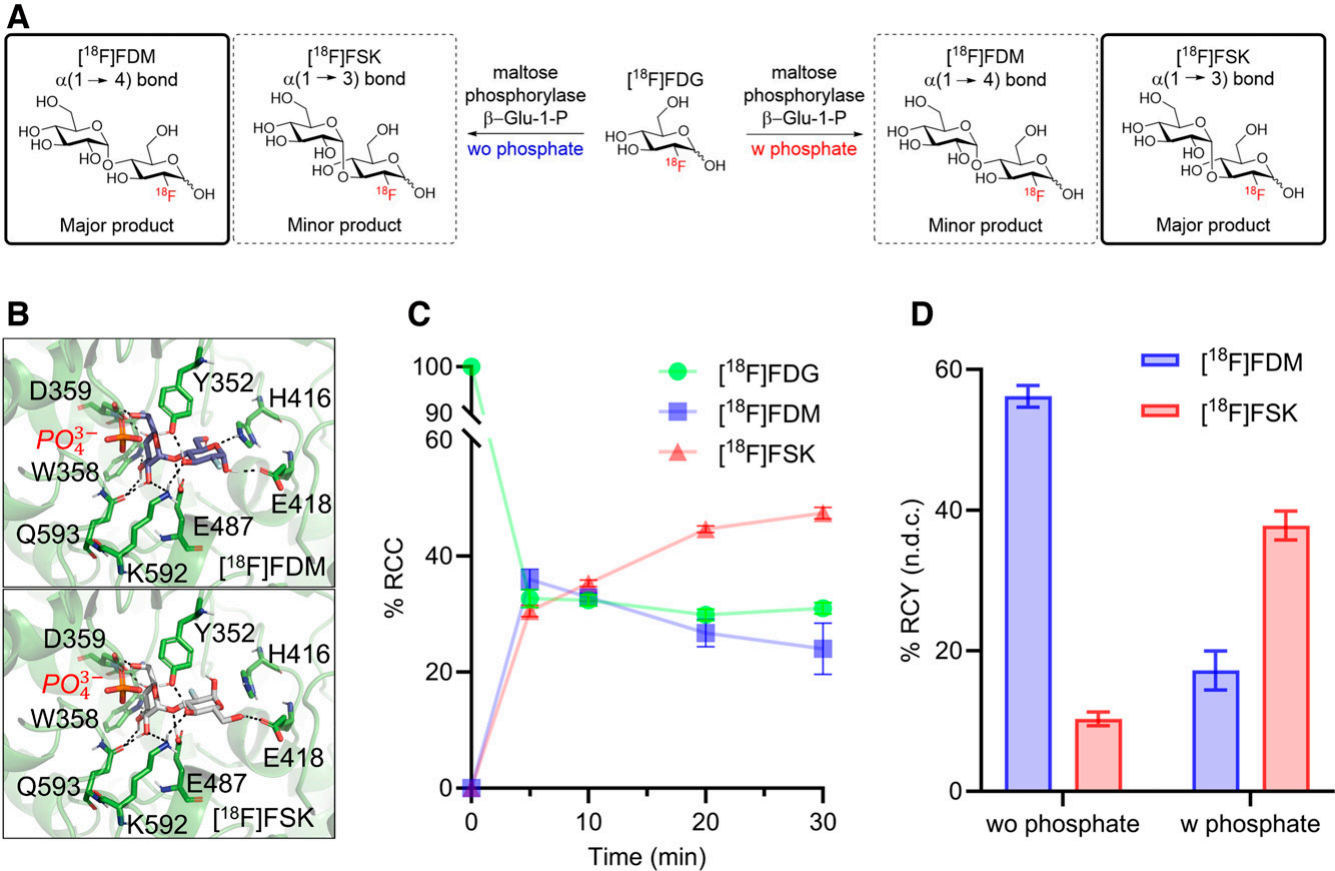
Improved radiochemical yield of [^18^F]FSK using MP. (A) Scheme of phosphate-modulated tunable reverse phosphorolysis of MP. (B) Top-scored docking poses returned by Molecular Mechanics/Generalized Born Surface Area simulations performed on [^18^F]FDM and [^18^F]FSK within binding site of MP (Protein Data Bank code: 1H54). Ligands and important residues are rendered as sticks and protein as cartoon. H-bonds are itemized by black dotted line. For sake of clarity, only polar hydrogen atoms are shown. (C) Radiochemical conversion (RCC) of [^18^F]FDG into [^18^F]FDM and [^18^F]FSK over time in presence of inorganic phosphate. (D) Comparison of isolated radiochemical yields (RCY) of [^18^F]FDM and [^18^F]FSK in different conditions. n.d.c. = non–decay-corrected; w = with; wo = without.

[^18^F]FSK was identified as the optimal [^18^F]FDG-derived sensor for detecting *S. aureus*. Therefore, we sought to improve its radiosynthetic yield. MP has strict substrate specificity (α-1,4 linkage) in phosphorolysis, using maltose but not nigerose as a substrate (34,35). Surprisingly, we found that [^18^F]FSK is obtained as a minor product in the MP-catalyzed reaction, likely because of the loss of enzyme specificity when the 2-position hydroxyl group of the “acceptor” sugar is absent (Fig. 4A; without phosphate). Density functional theory calculations suggest that α-1,4–linked products (maltose or FDM) are energetically more favorable than α-1,3–linked products (nigerose or FSK), which may support the observed preferential formation of [^18^F]FDM (Supplemental Fig. 5). Meanwhile, the top-scoring docking poses of [^18^F]FDM and [^18^F]FSK in MP (Supplemental Fig. 6) (15) showed that [^18^F]FSK binds less favorably (Molecular Mechanics/Generalized Born Surface Area binding free energy [*ΔG*] = −61.84 kcal/mol) than does [^18^F]FDM (*ΔG* = −68.24 kcal/mol), supporting its substrate specificity (Fig. 4B). In particular, [^18^F]FSK is not predicted to interact with H416. On the basis of this specificity, we hypothesized that [^18^F]FSK may serve as a poor substrate for forward direction phosphorolysis and that the addition of a phosphate source may promote phosphorolysis of the major product [^18^F]FDM via MP.

For this reason, chemoenzymatic radiosynthesis using MP with [^18^F]FDG and β-glucose-1-phosphate was performed in the presence of added inorganic phosphate and monitored over time (*n* = 3 for each time point) by radio–high-performance liquid chromatography (HPLC) (Fig. 4A; with phosphate). [^18^F]FDG was rapidly converted into [^18^F]FDM at early time points (36.8% ± 0.6% at 5 min), but the proportion of [^18^F]FDM gradually decreased over time (24.0% ± 4.4% at 30 min). Meanwhile, [^18^F]FSK was formed to 30.6% ± 0.8% at 5 min and gradually increased over time (47.4% ± 1.0% at 30 min) (Fig. 4C; Supplemental Fig. 7). The HPLC-isolated radiochemical yield was compared between the reaction with (*n* = 11) and without the additive (*n* = 3) (Fig. 4D; Supplemental Fig. 8). The addition of a phosphate source led to a significant increase in the yield of [^18^F]FSK (37.8% ± 2.0% vs. 10.3% ± 0.9% with and without the additive, respectively) while reducing the yield of [^18^F]FDM (17.2% ± 2.8% vs. 56.2% ± 1.6% with and without the additive, respectively). Although the addition of a phosphate source clearly altered the product ratio between [^18^F]FDM and [^18^F]FSK, the mechanistic basis for this observation was not fully investigated in this study. Future studies are needed to understand this phenomenon.

### Identification and Screening of NPs for α-1,3 Selective Radiosynthesis of [^18^F]FSK

**FIGURE 5.**
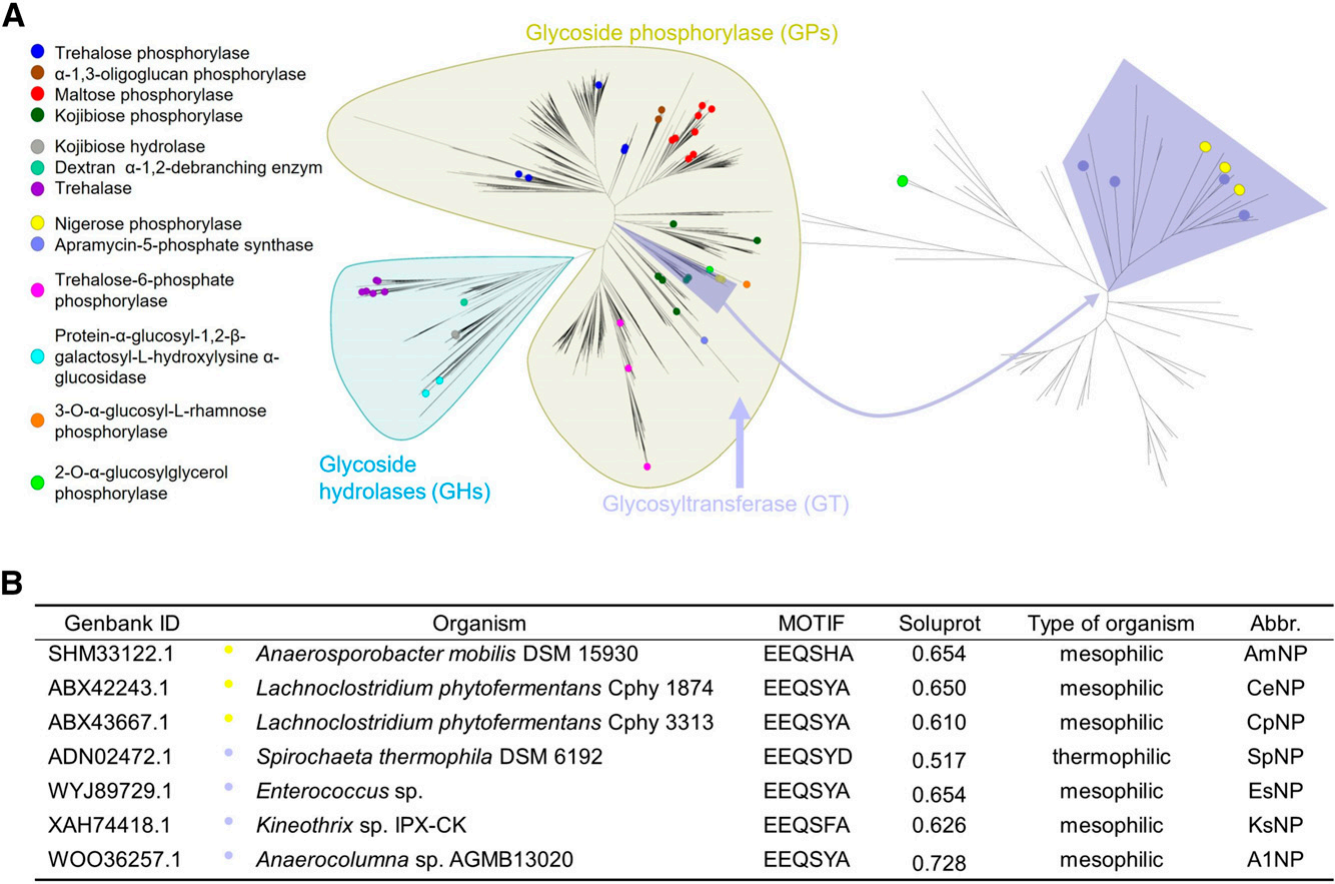
Identification of NP from GH65 family. (A) Phylogenetic tree from GH65 and pruned tree at node I3439 where NP branch is highlighted in blue: NPs already described in literature (yellow) and ones selected in this study (light blue). (B) Overview of NP found in GH65 family and selected in this study. Motif: consensus motif of 6 correlated positions (left to right positions: 64, 392, 394, 402, 416, and 585; *Caldicellulosiruptor saccharolyticus* kojibiose phosphorylase numbering).

Despite the improved MP-mediated radiosynthesis of [^18^F]FSK, residual [^18^F]FDG (the byproduct [^18^F]FDM) and natural abundance maltose remained a major chromatographic challenge. We therefore sought a method to produce [^18^F]FSK directly via an α-1,3–specific NP. To this end, the glycoside hydrolase family 65 (GH65) in the CAZyme database was explored to find new NPs with higher expression, activity, or stability. Before this study, a phylogenetic tree of GH65 was built by De Beul et al. (36,37) (Fig. 5A). De Beul et al. unveiled a network of 24 correlated positions, of which residues 64, 392, 394, 402, 416, and 585 (*Caldicellulosiruptor saccharolyticus* kojibiose phosphorylase numbering; consensus motif) showed the strongest evolutionary correlation, enabling the annotation of the phylogenetic tree on the basis of substrate specificity and dividing the GH65 family into 22 putative specificity subgroups, including an NP-specific branch (36,37). In total, 18 sequences were included in the NP branch, 3 of which are described in literature, namely NPs originating from *Anaerosporobacter mobilis* DSM 15930 (AmNP) (38), *Lachnoclostridium phytofermentans* Cphy 1874 (39,40), and *Lachnoclostridium phytofermentans* Cphy 3313 (CpNP) (40).

To predict which natural homologs would be suitable templates, their SoluProt score (an online tool to predict the soluble expression of proteins in *E. coli*) (41), organism of origin, and the consensus motifs of 6 correlated positions were compared. From all sequences, only 1 NP originated from a thermophilic organism (*Spirochaeta thermophila* DSM 6192 [SpNP]) and thus was selected. A selection of NPs originating from mesophilic species was also made on the basis of their consensus motif and highest SoluProt score (Fig. 5B). In total, 4 NPs originating from SpNP were selected: *Enterococcus* species (EsNP), *Kineothrix* species IPX-CK (KsNP), and *Anaerocolumna* species AGMB13020 (A1NP). Among the selected NPs, SpNP was expected to show robust and reproducible performance for future [^18^F]FSK production because of the high thermal stability of thermophilic enzymes. Therefore, SpNP was evaluated for thermal stability (versus the mesophilic CpNP), optimal temperature, optimal pH, and activity toward the fluorinated substrate FDG (nonradioactive). These studies showed that SpNP had high thermal stability, an optimal temperature of 70 °C (compared with −40 °C for CpNP) (40), and lower activity toward FDG (nonradioactive) versus glucose (Supplemental Fig. 9).

**FIGURE 6.**
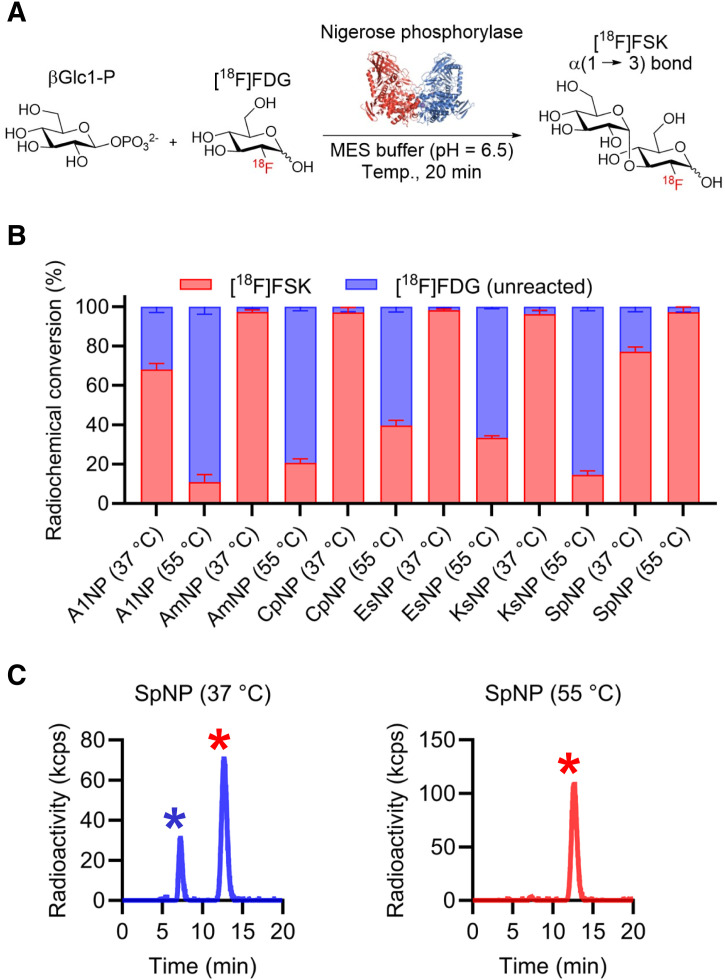
Improved radiosynthesis of [^18^F]FSK using thermostable SpNP. (A) Scheme NP-mediated chemoenzymatic conversion of [^18^F]FDG to [^18^F]FSK. (B) Comparative radiochemical conversion of [^18^F]FDG to [^18^F]FSK using different NPs at 37 °C and 55 °C. (C) Representative HPLC profiles of reaction using SpNP. kcps = kilocounts per second; MES = 2-morpholinoethanesulphonic acid.

Next, we performed comparative radiosyntheses of [^18^F]FSK using the 6 purified NPs (AmNP, CpNP, and the 4 newly identified NPs) at 2 temperatures (37 °C and 55 °C, *n* = 3 for each) (Fig. 6A). At 37 °C, most enzymes from mesophilic organisms (AmNP, CpNP, EsNP, and KsNP) showed quantitative conversion of [^18^F]FDG into [^18^F]FSK (>96%), whereas A1NP exhibited relatively low conversion yield (68.1% ± 3.0%). When these enzymes were screened at 55 °C, the yield of [^18^F]FSK dramatically decreased, from 10.9% to 39.6%. In contrast, SpNP showed a high rate of conversion of [^18^F]FDG to [^18^F]FSK at 37 °C (77.0% ± 2.5%) and 55 °C (97.2% ± 2.6%) (Figs. 6B and 6C). The isolated radiochemical yield of [^18^F]FSK using SpNP at 55 °C (71.0% ± 4.0%, *n* = 3, non–decay-corrected) was significantly higher than the yield using MP (Fig. 4D). Because NP cannot use 2-deoxy-glucose analogs for trisaccharide formation (34), no other radioactive byproducts were observed.

## DISCUSSION

In this study, we identified [^18^F]FSK as an optimal [^18^F]FDG-derived disaccharide to accurately detect *S. aureus*, a key pathogen in musculoskeletal and other infections. [^18^F]FSK showed high and consistent detection sensitivity in vitro in MRSA clinical isolates and identified the treatment response in a preclinical model of MRSA infection. [^18^F]FSK also showed low background signals in normal mice and had a favorable dosimetry profile. Before clinical translation, we sought a method to improve the radiosynthetic yield of [^18^F]FSK.

The discovery of [^18^F]FSK as a potent bacteria-specific sensor was previously facilitated by its radiosynthesis using commercially available MP (12). MP was previously shown to use a broad range of monosaccharides as acceptors in reverse phosphorolysis to generate the corresponding α-1,4–linked disaccharides (34,42). However, a recent study showed that α-1,3–linked disaccharides could be generated when 2-deoxy-d-glucose served as an acceptor, suggesting that an upside-down binding orientation of 2-deoxy-d-glucose to the subsite plus 1 allows the 3-hydroxyl group to function as the nucleophile during reverse phosphorolysis (35). Consistent with this mechanistic rationale, MP could also use [^18^F]FDG as an acceptor to generate [^18^F]FSK as a minor product. Although the role of added phosphate remains unclear, we found that the product ratio between [^18^F]FDM and [^18^F]FSK could be modulated by adding inorganic phosphate. This phenomenon may be attributable to varying rates of phosphorolysis, but it could also reflect differences in pH, ionic strength, and the potential effects on enzyme conformation.

Although most GH65 enzymes exhibit strict substrate specificity, previous studies demonstrate that NP can also generate or degrade both nigerose and kojibiose (37,39). Given that [^18^F]FDG is a 2-deoxy-glucose analog, the α-1,2–linked product (^18^F-kojibiose) cannot be formed, [^18^F]FSK is expected as a single product of reverse phosphorolysis via NP. On this basis, we expected that NP might be more suitable for selective and efficient radiosynthesis of [^18^F]FSK for future clinical translation. Our comparative evaluation indicated that SpNP exhibits superior thermal stability, enabling high [^18^F]FSK conversion at both 37 °C and 55 °C. Although the mesophilic NPs showed reduced enzymatic activity at 55 °C, these enzymes produced a higher yield of [^18^F]FSK than the alternative MP-based approaches. Because of its high thermal stability, we identified SpNP as the lead enzyme candidate for future automation of [^18^F]FSK. Moreover, its quantitative conversion of [^18^F]FSK allowed us to develop a simple and rapid method of cartridge-based purification to formulate an injectable [^18^F]FSK solution (Supplemental Fig. 10). One minor concern regarding an [^18^F]FDG-derived radiotracer is the presence of residual [^18^F]FDG (<2%) in the final solution (13,14). Using an optimized solid-phase extraction method (NH_2_-Plus; Sep Pak), we formulated [^18^F]FSK with high radiochemical purity (>99%) and undetectable [^18^F]FDG. With the goal of automation, future studies will aim to optimize the radiosynthesis of [^18^F]FSK by varying reagent concentration and further evaluating cartridge-based purification across a range of [^18^F]FDG molar activities.

One limitation of this study is that mice have high levels of α-glucosidase in the blood (12). This is particularly relevant for [^18^F]FDG-derived α-linked disaccharides, since their metabolic degradation may generate [^18^F]FDG, which may accumulate in host tissues or at sites of sterile inflammation (12,13). We previously demonstrated that coadministration of an α-glucosidase inhibitor (voglibose) enabled the in vivo evaluation of [^18^F]FSK and supported its ability to distinguish bacterial infection from inflammation (12). This approach is necessary to better approximate performance in patients. The high stability of [^18^F]FSK in human serum and liver microsomes supports its translational potential. First-in-human studies to evaluate the metabolism and pharmacokinetics of [^18^F]FSK will be performed without coadministration of an α-glucosidase inhibitor.

## CONCLUSION

The chemoenzymatic radiosynthesis of ^18^F-labeled oligosaccharides from widely available [^18^F]FDG represents a new paradigm in PET, potentially allowing the on-demand production of radiopharmaceuticals in the acute care setting. The identification and engineering of enzymes for this purpose is another new paradigm, as highlighted by our discovery of new NPs for the chemoenzymatic radiosynthesis of the bacteria-specific disaccharide [^18^F]FSK. In the future we anticipate that the integration of chemoenzymatic chemistry into radiosynthesis can allow the efficient and environmentally friendly construction of complex tracers with defined regiochemistry and stereochemistry to better characterize human disease.

## DISCLOSURE

This work was funded by the National Institutes of Health (R01EB030897, R01AI181378, R01AI161027, R21AI164684, R01EB028338, R01AI192221) and the National Supercomputing Center with supercomputing resources, including technical support (KSC-2025-CRE-0023). The μPET/CT scanner used for this research was acquired through an NIH High-End Instrumentation Grant (S10OD034286). No other potential conflict of interest relevant to this article was reported.

## Notes

KEY POINTS **QUESTION:** Can we harness the power of enzymes to conduct rapid, high-yield chemoenzymatic radiosyntheses of complex [^18^F]FDG-derived PET radiotracers with high regioselectivity and stereoselectivity? **PERTINENT FINDINGS:** We screened several α- and β-linked positron-labeled disaccharides obtained via phosphorolysis for their accumulation by the gram-positive organism *S. aureus*, identifying the α-1,3–derived tracer [^18^F]FSK as having the most robust performance in vitro and in vivo. The direct and efficient radiosynthesis of [^18^F]FSK was possible via a thermostable NP identified from a phylogenetic screen of GH65 enzymes. **IMPLICATIONS FOR PATIENT CARE:** In the future, numerous clinical PET radiotracers can be synthesized from the widely available synthon [^18^F]FDG, via chemoenzymatic radiosynthesis facilitated by the discovery or engineering of relevant enzymes.
